# Adaptive Intervention to Prevent Respiratory Illness in Cerebral Palsy: Protocol for a Feasibility Pilot Randomized Controlled Trial

**DOI:** 10.2196/49705

**Published:** 2024-01-08

**Authors:** Alyssa Fleischman, Carlos Lerner, Heidi Kloster, Paul Chung, Thomas Klitzner, Christopher Cushing, Danielle Gerber, Barbara Katz, Gemma Warner, Kristina Devi Singh-Verdeflor, Roxana Delgado-Martinez, Lorena Porras-Javier, Siem Ia, Teresa Wagner, Mary Ehlenbach, Ryan Coller

**Affiliations:** 1 Department of Pediatrics University of Wisconsin School of Medicine and Public Health Madison, WI United States; 2 Department of Pediatrics David Geffen School of Medicine at UCLA Los Angeles, CA United States; 3 Department of Health Systems Science Kaiser Permanente School of Medicine Pasadena, CA United States; 4 Clinical Child Psychology Program and Schiefelbusch Life Span Institute University of Kansas Kansas, KS United States; 5 Family Voices of Wisconsin Madison, WI United States; 6 UW Health Kids American Family Children's Hospital Madison, WI United States

**Keywords:** just-in-time adaptive intervention, respiratory illness, cerebral palsy, action planning, digital health

## Abstract

**Background:**

This study will pilot-test an innovative just-in-time adaptive intervention to reduce severe respiratory illness among children with severe cerebral palsy (CP). Our intervention program, Respiratory Exacerbation–Plans for Action and Care Transitions (RE-PACT), delivers timely customized action planning and rapid clinical response when hospitalization risk is elevated.

**Objective:**

This study aims to establish RE-PACT’s feasibility, acceptability, and fidelity in up to 90 children with severe CP. An additional aim is to preliminarily estimate RE-PACT’s effect size.

**Methods:**

The study will recruit up to 90 caregivers of children with severe CP aged 0 to 17 years who are cared for by a respiratory specialist or are receiving daily respiratory treatments. Participants will be recruited from pediatric complex care programs at the University of Wisconsin–Madison (UW) and the University of California, Los Angeles (UCLA). Study participants will be randomly assigned to receive usual care through the complex care clinical program at UW or UCLA or the study intervention, RE-PACT. The intervention involves action planning, rapid clinical response to prevent and manage respiratory illness, and weekly SMS text messaging surveillance of caregiver confidence for their child to avoid hospitalization. RE-PACT will be run through 3 successively larger 6-month trial waves, allowing ongoing protocol refinement according to prespecified definitions of success for measures of feasibility, acceptability, and fidelity. The feasibility measures include recruitment and intervention time. The acceptability measures include recruitment and completion rates as well as intervention satisfaction. The fidelity measures include observed versus expected rates of intervention and data collection activities. The primary clinical outcome is a severe respiratory illness, defined as a respiratory diagnosis requiring hospitalization. The secondary clinical outcomes include hospital days and emergency department visits, systemic steroid courses, systemic antibiotic courses, and death from severe respiratory illness.

**Results:**

The recruitment of the first wave began on April 27, 2022. To date, we have enrolled 30 (33%) out of 90 participants, as projected. The final wave of recruitment will end by October 31, 2023, and the final participant will complete the study by April 30, 2024. We will start analyzing the complete responses by April 30, 2024, and the publication of results is expected at the end of 2024.

**Conclusions:**

This pilot intervention, using adaptive just-in-time strategies, represents a novel approach to reducing the incidence of significant respiratory illness for children with severe CP. This protocol may be helpful to other researchers and health care providers caring for patients at high risk for acute severe illness exacerbations.

**Trial Registration:**

ClinicalTrials.gov NCT05292365; https://clinicaltrials.gov/study/NCT05292365

**International Registered Report Identifier (IRRID):**

DERR1-10.2196/49705

## Introduction

### Background

Children with severe cerebral palsy (CP) have spastic quadriplegia and are classified in level IV or V on the Gross Motor Function Classification System (GMFCS), often resulting in little or no independent mobility and serious respiratory consequences [1]. The mechanisms of respiratory illness in severe CP vary, paralleling those of other neuromuscular diseases [2], and include respiratory muscle weakness, recurrent infections and aspiration with inflammatory fibrosis, impaired airway clearance from altered tone, upper airway abnormalities, and poor chest wall compliance [3,4].

Respiratory illness is consistently the leading cause of death and hospitalization in severe CP [5,6]. Respiratory illness accounts for 59% of the deaths [5,7] and 25% of the hospitalizations [8-10] in severe CP. Moreover, respiratory illness strongly predicts future risk: respiratory hospitalization risk is 10-fold higher with a respiratory illness in the past year. Nevertheless, respiratory illness risk factors in severe CP are considered modifiable [11]. The prevention of these events is a significant need and a key to improving the quality of life and decreasing mortality [1,12].

Preventing hospitalization requires the opportunity for families and clinical teams to connect early enough to change trajectory [13-15]. Parents of children with CP have expressed the need for interventions focused on crisis management and self-efficacy [9,13,16]. However, respiratory illness in severe CP has broad comorbid triggers (eg, emesis, dysphagia, aspiration, and seizures). Because of this complexity, simple action plans or coaching alone may not address the breadth of respiratory illness triggers or potential responses; for example, if a parent of a child with severe CP follows an action plan directed toward bronchospasm, it would not effectively address an acute infectious lower respiratory infection. Parents of children with severe CP need comprehensive action planning and coaching; they also need an efficient direct extension to their clinical team for a just-in-time (JIT) adaptive clinical response directed specifically to acute real-time problems.

Currently, difficulty identifying when JIT care is needed is a barrier to effective illness response. Concerns may not reach clinical teams until an emergency department (ED) visit or hospitalization is inevitable. A national expert panel to identify interventions to prevent the hospitalization of children with complex diseases concluded that enhanced access, proactive crisis planning, and support for caregiver technical skills were crucial strategies to lower hospital use [17]. Prior postdischarge research has confirmed that admissions and ED visits could be better predicted by identifying when parents were not confident that their child with chronic conditions could avoid hospitalization or an ED visit than by other clinical or demographic indicators [18,19]. Preliminary work with a cohort that included children with severe CP demonstrated that parent confidence, monitored prospectively and repeatedly by SMS text message, is feasible, is acceptable, and predicts hospitalization within 2 weeks. This program of research will drive care forward by providing JIT care triggered by parents’ self-reported period of low confidence, thus matching the intervention to the immediate clinical need and preventing respiratory crisis.

### Prior Work

This team developed the earlier Plans for Action and Care Transitions (PACT) intervention to prevent hospitalizations for children with complex chronic diseases, including severe CP. After integrating a systematic literature review [20], parent interviews [21], and a national expert panel [17], each focused on preventing hospitalization, the team designed PACT to leverage evidence-based strategies from different populations: asthma action planning [22-24], health coaching [25-27], and feedback from parent advisory group meetings. The PACT intervention delivered action planning and coaching activities to children with diverse complex diseases, including severe CP, and observed 40% lower hospitalization rates for intervention versus control patients [28]. Simultaneously, our prior multisite research observed that confidence to avoid hospitalization, measured through repeated SMS text messaging, predicted hospitalization over the subsequent 2 weeks. Our clinical team and family partners hypothesized that periods of low confidence might be a useful tailoring variable to prompt intervention delivery [29].

The pact intervention has now been adapted to prevent severe respiratory illness in children with severe CP and to integrate SMS text messaging as a tailoring variable within a JIT adaptive intervention framework [29,30].

### Objectives

This pilot study (ClinicalTrials.gov: NCT05292365) is designed to establish the feasibility, acceptability, and fidelity of our intervention program, Respiratory Exacerbation–Plans for Action and Care Transitions (RE-PACT) in up to 90 children with severe CP and to establish a preliminary effect size of RE-PACT to inform a future efficacy study to reduce severe respiratory illness. This intervention consists of three related parts: (1) universal action planning, (2) an ongoing assessment of hospitalization risk, and (3) an algorithm to determine when to increase clinician contacts and tailor action plans. The study period will be divided into 3 waves; after each wave, feasibility, acceptability, and fidelity data will be reviewed against predefined measures of success to adjust the protocol and overcome implementation barriers. We describe the design and protocol of this trial in the following sections.

## Methods

### Participants and Setting

This intervention will recruit primary caregivers of children with severe CP. Up to 90 caregivers of children with severe (GMFCS level IV or V) CP aged 0 to 17 years and cared for by a respiratory specialist or receiving daily respiratory treatments will be enrolled. Participants will be recruited from pediatric complex care programs at the University of Wisconsin–Madison (UW) and the University of California, Los Angeles (UCLA). These programs were established to deliver care to children with medical complexity. The key components of each program include pediatric clinicians, care coordinators, and extended visit lengths, which aid in delivering comprehensive care to children with CP. Both clinical programs have been described in more detail elsewhere [28,31].

### Inclusion Criteria

Participants are caregivers of children with severe CP. Individuals must meet all inclusion criteria to be eligible to participate in the study. Caregiver criteria include (1) being aged at least 18 years, (2) being the primary caregiver to an eligible child, (3) ability to speak English or Spanish well enough to be interviewed, and (4) having a mobile phone capable of sending and receiving SMS text messages. Child criteria include (1) age 0 to 17 years, (2) GMFCS level IV or V CP [32], and (3) being cared for by a respiratory specialist or receiving daily respiratory treatments (oxygen, ventilation, airway clearance device, and medications).

### Exclusion Criteria

During this study, participants are asked to reply to SMS text messages when received at random times during daytime hours and connect with an intervention clinical responder either at home, in person at a mutually agreeable location, by mobile phone, or over the internet. Any individual lacking the ability or willingness to engage in SMS text messaging or clinical responder interactions during the study will be excluded from participation in the study.

### Recruitment and Screening

We will recruit caregivers of children with severe CP aged between 0 and 17 years. We will recruit up to 90 participants (n=45, 50% at each site) divided across 3 waves. In each wave, there is a 1- to 2-month enrollment period. We anticipate that approximately 80% of those screened will enroll, requiring approximately 110 individuals to be screened.

Using diagnostic codes for CP (International Classification of Diseases, Tenth Revision [ICD-10]: G80-83), we will identify potential participants by reviewing clinic registries and electronic health record data, which contain detailed information about children and their diagnoses. We will send an *opt-out* letter that alerts families that a research study is being conducted and their child may be eligible, with a contact number to call if they wish to opt out of the research or if they wish to receive additional information or have any questions. Potentially eligible caregivers will be contacted by telephone to screen for eligibility and interest.

If the research team is not notified that a family wishes to opt out of the research, the study research personnel will attempt to call the families (or meet them at an upcoming visit) to complete screening, informed consent, baseline questionnaires, and random group assignment. CP status and additional eligibility criteria will be determined with a reliable and valid parent questionnaire and screener conducted at the beginning of the initial telephone contact [32].

Individuals who do not meet the criteria for participation in this trial (screen failure) because they meet ≥1 exclusion criteria that are likely to change over time may be rescreened. Theoretical examples might include a child developing a need for respiratory treatment or families acquiring a mobile phone capable of sending and receiving SMS text messages.

All study participants will undergo informed consent, including authorization to view the child’s medical record and participate in action planning, rapid clinical response, and weekly SMS text message surveillance.

### Study Design

This is a 2-site pilot randomized controlled clinical trial to establish the RE-PACT protocol’s feasibility, acceptability, and fidelity as well as an estimate of effect size. We anticipate being underpowered to assess the efficacy of the intervention in this pilot study; however, to inform future randomized controlled trial power estimates, we will test differences between the intervention and control groups in primary and secondary clinical outcomes.

Study participants will be randomly assigned to receive usual care through the complex care clinical program at UW or UCLA or the study intervention, RE-PACT. Random allocation will be concealed from the research staff conducting recruitment and will use a 1:1 allocation with random block sizes of 2 and 4. Block randomization will be achieved with a computer-generated random number list prepared by the study biostatistician without clinical involvement in the trial. Randomization will be stratified by site to account for site-specific study characteristics.

RE-PACT will be run through 3 successively larger 6-month trials (*waves*), allowing ongoing protocol refinement between waves, guided by prespecified definitions of success for feasibility, acceptability, and fidelity measures. Each wave has a specific protocol refinement focus (wave 1: onboarding, training, recruitment, and data collection; wave 2: randomization and intervention activities; and wave 3: rapid enrollment and the conduct of all protocol activities with high fidelity). Participants in both groups will undergo assessments of demographic, clinical, and caregiving measures using questionnaires and medical record review case report forms at baseline and at 6 months after enrollment. Intervention feasibility, acceptability, and fidelity data will be collected from parent reports, medical records, and research team logs using case report forms.

### Description of the Intervention

RE-PACT uses a dynamic JIT adaptive intervention design [33] to deliver proactive intervention based on risk modeling and partnership between the care team, patients, and families. Although the causes of respiratory illness in severe CP are modifiable, they are also broad and require distinct responses, even for the same child, over time. RE-PACT assumes that (1) every patient with severe CP has a risk of hospitalization, (2) some risks are knowable via the ecosystem of data generated around patient care, and (3) an intervention delivered when risk is increasing can reduce hospitalizations. RE-PACT’s design addresses the changing needs of a child and family. RE-PACT involves action planning, rapid clinical response to prevent and manage respiratory illness, and weekly SMS text messaging surveillance of caregiver confidence for their child to avoid hospitalization.

### Action Planning

#### Overview

All intervention families will receive respiratory illness action plans within 1 month of study entry. The action plan format and process are adapted from the original PACT study, and the contents include (at minimum) recognizing, describing, and managing the child’s known contributors to respiratory illness. The three main components of the action plans are (1) *focus area* for the action plan (eg, asthma, aspiration, and seizures); (2) *severity levels* corresponding to objective and subjective indicators of baseline (*green*), concerning (*yellow*), and severe (*red*) statuses (eg, >2 L/min of oxygen); and (3) *specific actions* that caregivers should take to manage each status (eg, increase vest therapy, albuterol, suction every 4 hours, and use oxygen up to 4 L/min). As needed, JIT plans are also created at times of low confidence by parent request or by clinician determination during the study period. Any plan created will be developed with families, target an issue that plausibly will recur and lead to respiratory illness–related ED or hospital visit, and, when relevant, be harmonized with prior plans and reflect pulmonologist agreement.

#### Mobile Health Platform

**Figure 1:**
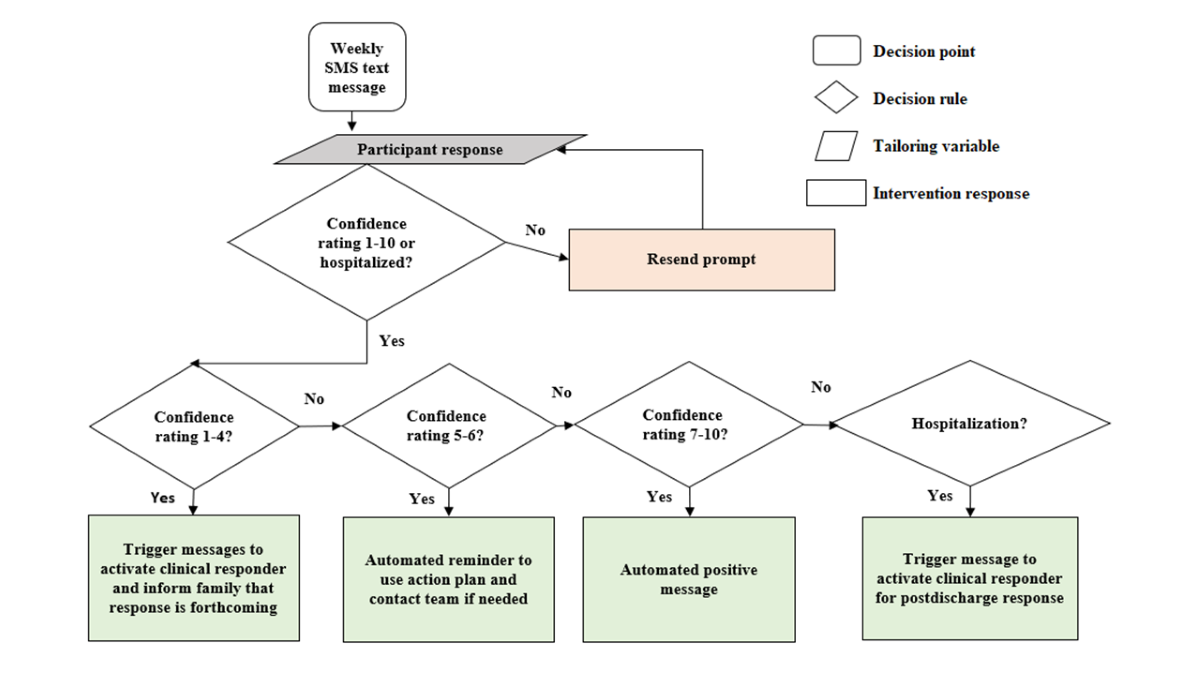
Schematic of the mobile health (mHealth) SMS text message process in which all study participants receive weekly SMS text messages asking them to rate their confidence for their child to avoid hospitalization in the next month.

The mobile health (mHealth) platform is built from an earlier study, *Assessing Confidence at Times of Increased Vulnerability* (ACTIV) [29], which was designed to elicit a SMS text rating of confidence to avoid hospitalization in the next month (ratings range from 1 to 10, where 1 is lowest confidence, and 10 is highest confidence; Figure 1). The platform supports English and Spanish languages. Beginning on the Sunday after enrollment, families will start receiving weekly SMS text messages asking them to rate their confidence for their child to avoid hospitalization in the next month. SMS text messages are programmed to be sent at random days and times to caregivers, averaging once weekly (Sunday to Thursday) between 8 AM and 9 PM (local time). The Sunday-to-Thursday time frame was chosen to support a feasible response during business days and hours. After 2 hours of nonresponse, a reminder is sent, and this is repeated up to 2 times at 2-hour intervals. Clinical responders will receive an email notification in real time if a participant reports low confidence. In addition, clinical responders will receive an SMS text message notification between 9 AM and 6 PM with the report of low confidence. If a response comes outside of these hours, it will be delayed until the next day.

#### Rapid Clinical Response

**Figure 2:**
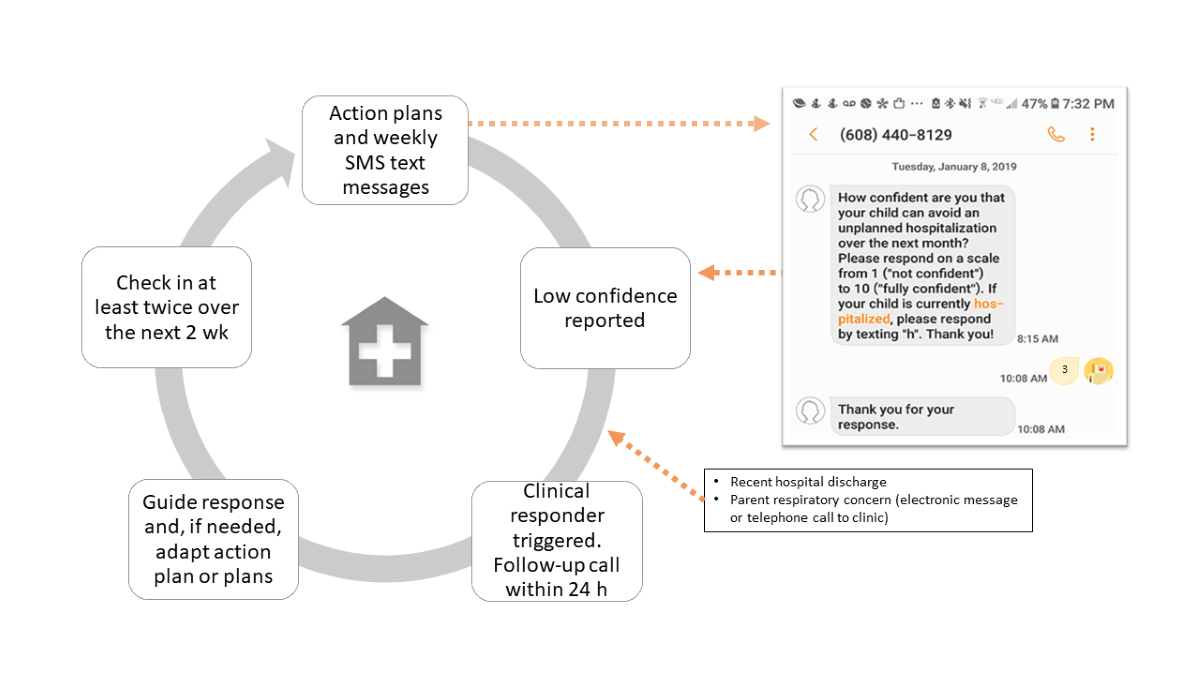
Summary of the Respiratory Exacerbation–Plans for Action and Care Transitions (RE-PACT) intervention. The figure illustrates low-confidence SMS text messages as the trigger of rapid clinical response. Other triggers include hospital discharge or family-expressed respiratory concerns through telephone call or electronic message to the clinic.

This response is adapted from our prior intervention (PACT) [28]. In RE-PACT, a clinical responder guides the JIT response, adapting to the current child and family situation. Triggers for the clinical responder include (1) low family-reported confidence (a confidence rating of <5) during mHealth messaging, (2) hospital discharge, and (3) family call or electronic message to the clinic owing to acute respiratory concerns (Figure 2). Clinical responders are clinicians, including medical doctors, nurse practitioners, registered nurses, and care coordinators (or equivalent). The same responder intends to work with the family throughout study enrollment.

**Figure 3:**
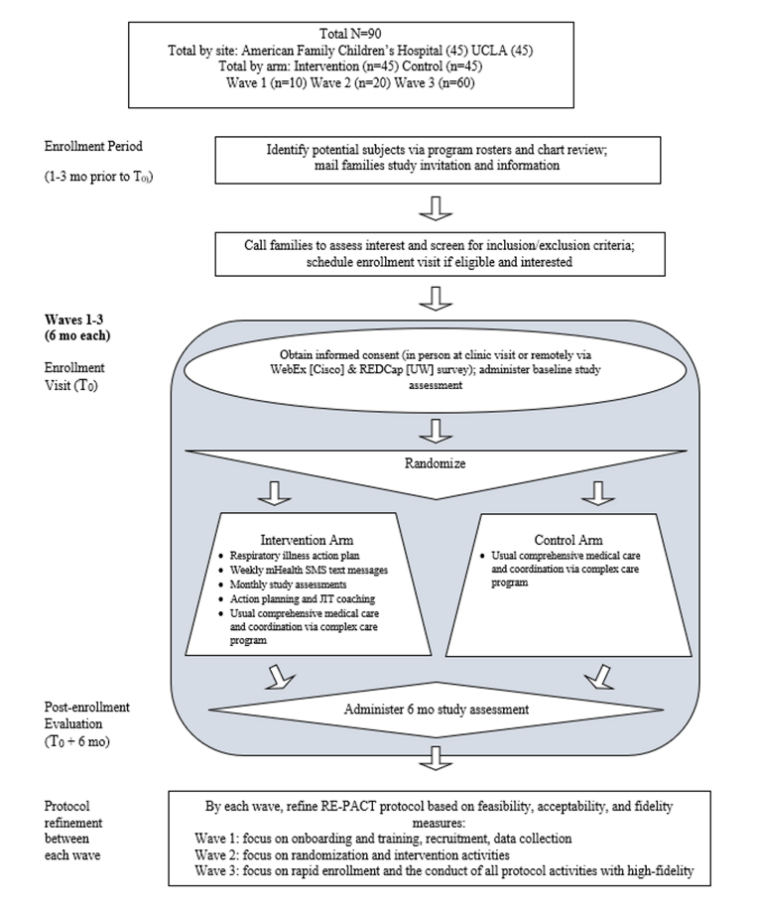
Schematic of the study design of Respiratory Exacerbation–Plans for Action and Care Transitions (RE-PACT) intervention enrollment period, enrollment visit, postenrollment evaluation, and protocol refinement. The protocol is refined between each of the 3 waves of the RE-PACT study. JIT: just-in-time; mHealth: mobile health; REDCap: Research Electronic Data Capture; UCLA: University of California, Los Angeles; UW: University of Wisconsin–Madison.

Rapid clinical responses include 3 interactions between family participants and clinical responders. First, a triage contact occurs within 24 hours of a trigger (during business hours). The triage contact goals are to determine the nature of the trigger, whether an action plan exists for the situation, and whether the issue is within the clinical responder’s clinical practice scope. If not within the scope, the issue is referred to the relevant support (eg, a specialty physician or clinic social worker). Second, a response planning visit occurs either as a component of the triage contact or at a mutually agreed upon time within 72 hours of the trigger. Third, at least 2 follow-up contacts occur within 2 weeks of the trigger, with additional follow-ups as indicated by ongoing need until the issue is resolved. All contacts can occur through any of the following, at the preference of the family: telephone call, clinical encounter (telehealth, clinic, and hospitalization), or a home visit. The follow-up contacts can occur through electronic communication if the clinical responder and family determine this to be appropriate. At each contact point, there are two goals: (1) ensuring that the family understands red flags, relevant medications, and whom to call and when and keeps notes about the issue; and (2) coaching and skill transfer for the family to generate solutions and lead actions, with the responder intervening if the family is stuck or if clinical needs dictate intervention. Each contact point has scripting to guide the clinical responder as well as electronic health record documentation templates. At the end of a clinical responder event, the responder determines whether the issue affects respiratory health, is likely to recur, and poses a risk for future ED or hospital visits. If all of these are true, the responder either updates existing action plans or creates a new one to address the issue. A participant is considered to have completed the study if they have completed the baseline and 6-month follow-up assessments (Table 1; Figure 3).

**Table 1:** Schedule of activities of the Respiratory Exacerbation–Plans for Action and Care Transitions (RE-PACT) intervention throughout the study period, with the depiction of personnel involved.

Activities			Study period (personnel involved)														
			Enrollment visit (research coordinator)		RE-PACT intervention period (clinicians)												Final visit (research coordinator)
			0 (T_0_)^a^		1 (T_1_)^a^		2 (T_2_)^a^		3 (T_3_)^a^		4 (T_4_)^a^		5 (T_5_)^a^		6 (T_6_)^a^		End of month 6 (T_7_)^a^
Confirm eligibility			✓														
Informed consent			✓														
Baseline assessment			✓														
6-mo assessment																	✓
Randomization			✓														
Participant compensation			✓														✓
Usual comprehensive medical care and coordination via complex care program					✓		✓		✓		✓		✓		✓		
**Intervention arm only**																	
	SMS text message training	✓															
	Weekly mHealth^b^ text message and response			✓		✓		✓		✓		✓		✓			
	Intervention overview			✓													
	Create action plan			✓													
	Action planning			✓		✓		✓		✓		✓		✓			
	Rapid clinical response when triggered			✓		✓		✓		✓		✓		✓			
	Monthly study assessments			✓		✓		✓		✓		✓		✓			

^a^Month (time point).

^b^mHealth: mobile health.

### Outcomes

#### Primary Study End Points: Feasibility, Acceptability, and Fidelity

The specific measures and prespecified definitions of success for primary study end points, including feasibility, acceptability, and fidelity, are listed in Table 2. These measures will be summarized between each of the 3 waves, with protocol adjustments made for any measures that do not meet the definition of success.

**Table 2:** Primary study end points to evaluate the feasibility, acceptability, and fidelity of Respiratory Exacerbation–Plans for Action and Care Transitions (RE-PACT; n=90).

Measure and measure detail				Success definition
**Feasibility**				
	Recruitment	Days to enroll target, mean (SD)	<14 (1)	
	Intervention onset	Days between randomization and “T_0_a^a^” intervention activities, mean (SD)	<7 (1)	
	Time to action plan	Days to action plan creation	<30	
	Intervention time	Time logged (min) for action planning and for coaching activities, mean (SD)	N/A^b^	
	Intervention costs	Mileage and travel costs; personnel salary; training costs; and other incurred costs, total	N/A	
	Intervention triggers	Number per patient (annualized), both respiratory and nonrespiratory focused	N/A	
**Acceptability**				
	Enrollment	Enrollment rate (number of patients enrolled/number approached)	>80%	
	Consent refusal	Categorized reasons for refusal	N/A	
	Loss and dropouts	Dropout rate (active or passive) before 6 mo (number of dropouts/number enrolled)	<10%	
	Action plan, SMS text messaging and clinical responder satisfaction	Do caregivers use the action plan, coaching, and texting? How could it be improved? Would caregivers recommend this to another family?	N/A	
**Fidelity**				
	Enrollment duration	Time (mo) of participant enrollment in the study, mean (SD)	6 (1)	
	Action plan creation	Number of respiratory and overall action plans per patient and action plan focus areas	≥1	
	Rapid clinical response: home or web-based visit	Success rate (number of visits completed and number expected); stratify by trigger and by “respiratory” and “nonrespiratory”	>80%	
	mHealth^c^ texting	Response rates (number of SMS texts responded and number expected); “respiratory” and “nonrespiratory”	>90%	
	Crossover	Number of patients inappropriately receiving any intervention component	0	
	Data collection	Complete entry and exit questionnaire, monthly questionnaire, and chart review data (number of data collection events completed and number of total data collection events)	>95%	

^a^a: Enrollment visit

^b^N/A: not applicable.

^c^mHealth: mobile health.

#### Clinical End Points

The study’s secondary objective is to estimate the effect size of RE-PACT. The clinical end points are listed in Textbox 1. The primary clinical end point is severe respiratory illness, defined as a respiratory diagnosis requiring hospitalization. *Respiratory diagnosis* is defined as a discharge diagnosis of any of the following: asthma, pneumonia (community or hospital acquired), bronchiolitis, influenza, upper or lower respiratory tract infection, tracheitis, aspiration pneumonia and pneumonitis, chronic lung disease, and respiratory failure [34]. *Hospitalization* is defined as a nonelective, unscheduled hospital encounter (inpatient or *observation* status), accompanied by both an admission history and physical examination as well as a discharge summary note signed by a physician or advanced practice provider. Field-testing the assessment of this end point with trained research personnel at study sites demonstrated high interrater reliability (κ>0.9).

**Clinical end points of Respiratory Exacerbation–Plans for Action and Care Transitions (RE-PACT).**

**Primary clinical end point**

- Severe respiratory illness, defined as a respiratory diagnosis requiring hospitalization

**Secondary clinical end points**

- Hospital days during severe respiratory illness
- Systemic steroid courses (systemic steroids [exclude inhaled or topical steroids for the purposes of defining an illness]: hydrocortisone, prednisone, prednisolone, dexamethasone, methylprednisolone, and triamcinolone acetonide; common inhaled steroids: fluticasone, budesonide, mometasone, beclomethasone, and triamcinolone [14-16,35])
- Systemic antibiotic courses (antibiotics: amoxicillin or amoxicillin/ and clavulanate, ampicillin, ampicillin and sulbactam, azithromycin, cefdinir, cefepime, cefixime, cefpodoxime, ceftazidime, ceftriaxone or cefotaxime, ceftibuten, cefuroxime, cephalexin [Keflex], clarithromycin, clindamycin, ciprofloxacin, doxycycline, erythromycin, ertapenem, imipenem, levofloxacin, linezolid, meropenem, metronidazole, moxifloxacin, oseltamivir, penicillin, piperacillin and tazobactam, rifampin, and vancomycin [14-16,35])
- Respiratory emergency department visits
- Death

The secondary clinical outcomes (Textbox 1) include total hospital days during severe respiratory illness; the number of systemic steroid courses, systemic antibiotic courses, and respiratory ED visits; and death. Hospital days are calculated through resolution if admission occurs in the study time frame, even if discharge occurs after the study exit date. *Systemic corticosteroid course* is defined by oral or parenteral corticosteroids prescribed for respiratory diagnosis, including hydrocortisone, prednisone, prednisolone, methylprednisolone at least 1 mg/kg/d (or 30 mg/d) × minimum 3 days, or dexamethasone at least 0.15 mg/kg/d (or 10 mg/d) × ≥1 days. Physiologic or stress replacement doses in adrenal insufficiency are excluded. *Systemic antibiotic course* is defined by oral or parenteral antibiotics prescribed for respiratory diagnosis for a minimum of 3 days. The specific antibiotics are derived from the Infectious Diseases Society of America pediatric pneumonia guidelines [36] and published literature [35]. Respiratory ED visits are any ED visits not resulting in admission and having a discharge respiratory diagnosis.

#### Exploratory Study End Points

The objectives of the tertiary study are to explore the mediating relationships between RE-PACT and capability, opportunity, motivation, and behavior (COM-B) measures [37]. By blending our foundational research on preventing hospitalizations [17,21] with behavioral intervention theory [38], our conceptual model suggests that decisions to seek care (behaviors) are influenced by capability (family capacity), opportunity (health system and susceptibility), and motivation (confidence). A theorized mechanism of RE-PACT’s effect is that combining action planning, mHealth surveillance, and coaching will increase caregiver COM-B measures to manage respiratory illness in severe CP. The tertiary end points are listed in Textbox 2.

**Exploratory study end points of Respiratory Exacerbation–Plans for Action and Care Transitions (RE-PACT).**

**Capability**

- Family Caregiver Activation in Transition (FCAT) tool [39]: mean composite score
- Caregiver General Self-Efficacy Scale (GSES) [40]: mean composite score

**Opportunity**

- Family Experiences with Care Coordination (FECC) [41]: percentage top-box score for selected items

**Motivation**

- Confidence responses mobile health SMS text messaging: mean weekly score

### Assessment Procedures

Data about research participants (children and their families) will be collected by study research assistants on case report forms using electronic family self-administered questionnaires, structured interviews with research personnel by telephone or in person with enrolled caregivers, and abstraction of child medical record data. Family and child measures will be recorded at baseline, and end points will be recorded at study exit (6 months after the enrollment visit [T_0_]). Caregiving measures, which may change as a result of the intervention, will be collected at baseline and study exit. Clinical responders will enter data for intervention group families into a clinical response event case report form.

Feasibility, acceptability, and fidelity end point data will be collected during each of the 3 waves by research personnel reviewing study logs, conducting monthly chart reviews, and administering surveys (by telephone, in person, or sending electronic self-administered links) with caregivers randomized to the intervention. For control group participants, the feasibility of assessments will be evaluated by completion rates at study exit. In addition, intervention and control participants will be debriefed at study exit on their experiences in the study and asked for feedback on the strengths and weaknesses, as well as any concerns about the protocol. Between each wave and after the third wave, clinical teams at each site will be debriefed on the strengths and weaknesses, as well as concerns about the protocol.

The CP GMFCS measures and all caregiving measures have been well documented as reliable in the literature [39-45]. We have separately established the reliability of identifying respiratory illnesses in our preliminary research (κ>0.9). We will ensure reliability in data collection through direct observation, data auditing, establishing clear data dictionaries and definitions, using uniform variable definitions, and use of a central data repository coordinated and maintained by UW.

### Data Collection, Storage, and Protection

Clinical data (including adverse effects) and clinical laboratory data will be entered into Research Electronic Data Capture (REDCap; UW) managed by the University of Wisconsin, a 21 Code of Federal Regulation Part 11–compliant data capture system provided by the UW Institute for Clinical and Translational Research. The data system includes password protection and internal quality checks, such as automatic range checks, to identify inconsistent, incomplete, or inaccurate data. Clinical data will be entered directly from the source documents or entered directly from secure self-administered questionnaires (surveys) sent via REDCap to participants.

### Sample Size Considerations

We will enroll up to 90 participants. On the basis of this team’s preliminary work, we estimate that half (45/90, 50%) of the participants will experience at least 1 respiratory illness during the enrollment period. We expect to be able to maintain contact and collect data from ≥90% (≥81/90) of the participants at the final follow-up, evenly divided between the intervention and control groups. We assume that this sample will not be powered to establish the efficacy of the intervention; however, it will provide a sufficient sample to determine feasibility and estimate effect sizes, which will be used for power calculations in the future large randomized clinical trial. Attainable power levels were calculated for detecting differences in severe respiratory illness rates (primary clinical outcome) between the study arms at the 2-tailed <.05 significance level based on a negative binomial (NB) regression model with an overdispersion parameter of φ=1.0 (Table 3). Hence, large effect sizes with relative risks ranging between 3.0 and 5.0 for comparing the severe respiratory illness rates between the study arms will be detected with 19% to 88% power at the 2-tailed <.05 significance level.

**Table 3:** Attainable power levels for detecting differences in severe respiratory illness rates between the study arms, assuming a sample size of 45 participants per arm with a missing value rate of ≤10%.

Relative risk (control vs intervention)	Number of severe respiratory illnesses in the intervention arm over the 6-mo follow-up period				
	5 (λ=0.02)^a^, %	10 (λ=0.40)^a^, %	15 (λ=0.06)^a^, %	20 (λ=0.08)^a^, %	25 (λ=0.10)^a^, %
3.0	19	29	38	45	52
4.0	31	41	59	68	75
5.0	42	62	74	83	88

^a^Severe respiratory illness rate per patient-month.

### Statistical Analysis Plan

The primary outcome data will assess the RE-PACT intervention’s feasibility, acceptability, and fidelity using descriptive statistics. Categorical variables will be displayed as percentages and continuous variables as means with SDs (if normally distributed) or medians with IQRs (if skewed). We will compare observed values with the prespecified definitions of success for each of our feasibility, acceptability, and fidelity measures. We will also determine overall positive, neutral, and negative reports of feasibility and acceptability using content analysis of qualitative (open-ended comments) data. We will explore any patterns if challenges emerge (eg, enrollment refusal or dropout or low reported use of the intervention activities).

We anticipate being underpowered to assess efficacy using the clinical end points of the intervention in this pilot study. Analyses will also estimate the effect size estimates of clinical end points to allow precise sample size calculations for a future large-scale efficacy trial. We will compare differences between the intervention and active control group outcomes at 6 months. The primary clinical outcome is the severe respiratory illness rate, defined as the total number of severe respiratory illnesses divided by the person-months over the 6-month follow-up period. The severe respiratory illness rate will be analyzed using an NB regression model to account for overdispersion in the count data. For the primary analysis, univariate NB regression analysis will be conducted with a study arm as a predictor variable. The study site will be included as a stratification factor in the primary analysis to account for stratified randomization. The observed effect size of the analysis will be quantified in terms of relative risk and reported along with the corresponding 95% CI. As a secondary analysis, multivariate NB regression analysis will be performed to compare the severe respiratory illness rates between the study arms. This analysis will include clinical and demographic characteristics as covariates in an initial nonparsimonious model. The least absolute shrinkage and selection operator and elastic net penalty methods for NB regression models will be used to identify a parsimonious model with independent covariates.

Longitudinal changes in the severe respiratory illnesses within and between study arms will be evaluated with a generalized linear mixed effects model with a logit link function and participant-specific random effects. An autoregressive correlation structure will be used to account for within-participant correlations. In this analysis, the presence or absence of severe respiratory illness at the monthly assessments will be the dependent variable, the study arm will be included as a predictor variable, and the study site will be included as a stratification variable to account for the stratified randomization.

The secondary clinical outcomes include total hospital days during severe respiratory illness; the number of systemic steroid courses, systemic antibiotic courses, and respiratory ED visits; and death. The number of systemic steroid courses, systemic antibiotic courses, and respiratory ED visits over the 6-month follow-up period will be analyzed using NB regression analysis as described previously for the primary outcome. Observed effect sizes and the corresponding 95% CIs will be reported. The presence or absence of systemic steroid courses, systemic antibiotic courses, and respiratory ED visits will be documented at the monthly assessments, and longitudinal changes within and between study arms will be analyzed using generalized linear mixed effects modeling with a logit link function and patient-specific random effects. The total number of hospital days over the 6-month follow-up period will be analyzed using ANOVA with the study site as a stratification factor. In a secondary analysis, an analysis of covariance (ANCOVA) will be performed where clinical and demographic baseline characteristics will be included as covariates, and the least absolute shrinkage and selection operator method will be used to identify a parsimonious model. Longitudinal changes in the number of hospital days per hospitalization will be analyzed using a normal mixture linear mixed effects model with patient-specific random effects. The normal mixture component will be included in the model to capture the probabilities of hospitalization at the monthly follow-up. Parameter estimation will be performed using the expectation-maximization algorithm, the standard method for the parameter estimation of mixture models.

Two-tailed *P* values of <.05 will be considered statistically significant. Missing values (eg, owing to loss of follow-up and missing monthly visits) will be evaluated by conducting a sensitivity analysis comparing the results obtained from the complete case analysis with those obtained from imputation-based analyses. Specifically, multiple imputations will be used to impute the missing values of the primary and secondary clinical outcomes. For monotonic missing value data structures, we will use regression-based multiple imputation techniques. By contrast, we will use Markov Chain Monte Carlo–based imputation techniques for nonmonotonic missing value data structure.

Although we anticipate that the intervention and control groups will be similar owing to random assignment, we will adjust for any variables in our analysis that are not equal between the groups, given the small sample size. In addition, we will analyze for any effect of primary home language on the study outcomes because this may affect families’ ability to navigate the systems of care in the United States.

Finally, as a planned exploratory analysis, we will test the mediating effect of caregiver COM-B measures on the relationship between intervention and respiratory illness outcomes. The mediating effects will be evaluated by conducting a multistep analysis approach. In the initial step, NB regression analyses will be conducted to examine whether there are differences in respiratory illness outcomes (the number of severe respiratory illnesses, systemic steroid courses, systemic antibiotic courses, and respiratory ED visits) between the intervention and control arms. In the next step, we will conduct a sequence of univariate analyses by regressing each potential mediator variable (caregiver capability COM-B measures) on the binary study arm variable. If significant associations between the potential mediator variables and the study arm are detected, we will regress the respiratory illness outcomes on both the mediator variables and study arm indicator variables using ANCOVA. The mediation effect for each potential mediator variable will then be tested using the Sobel *z* test based on the slope parameter estimates from the corresponding regression models.

### Ethical Considerations

This study received initial approval from the UW health system’s institutional review board on January 19, 2022 (20211532). All participants will provide informed consent before taking part in the study. Informed consent materials will be provided in private spaces in both written and verbal formats and will review in detail the study design, including random assignment to the intervention and control groups, potential risks of participation, protections against risk, and the rights of human research subjects. The informed consent process will also include review and signing of the Health Insurance Portability and Accountability Act waiver, allowing researchers to review the child’s medical records. Parents will be able to decline parts of the study and still participate in other parts and can revoke their consent at any point. Any identifying information kept for the purpose of contacting participants will be kept secure, in REDCap, a locked filing cabinet or in a password-protected electronic file and will be destroyed when the study is complete. The study is monitored by the Data Monitoring Committee at the UW-Madison Institute for Clinical and Translational Research. All participants receive an incentive of US $200, divided in 2 parts: US $100 at enrollment and US $100 after the exit survey, in the form of a gift card, check, or cash.

## Results

The recruitment of the first wave began on April 27, 2022. To date, we have enrolled 30 (33%) out of 90 participants, as projected. The final wave of recruitment will end by October 31, 2023, and the final participant will complete the study by April 30, 2024. We will start analyzing the complete responses by April 30, 2024, and the publication of results is expected at the end of 2024.

## Discussion

### Summary

We describe the protocol for a pilot clinical trial of RE-PACT, a JIT adaptive intervention to reduce respiratory illness in severe CP. A recent expert consensus statement on preventing and managing respiratory disease in young people with CP highlighted the need for 4 activities: early identification of risk factors; regular assessment of risk; effective partnerships among multidisciplinary teams, families, and individuals with CP; and proactive treatment of respiratory disease [46]. The RE-PACT intervention protocol aligns with each of these 4 critical areas.

For children with severe CP, RE-PACT was designed by families and clinicians from promising earlier interventions to manage health crises with proactive action planning, simple surveillance of family confidence to avoid hospitalization through frequent SMS text messaging, and JIT adaptive rapid clinical responses. This intervention breaks down barriers to equitably connect families and clinical teams precisely when it matters most. This approach is innovative because we tailor the intensity of the response (eg, telephone call and clinic visit) and its content to family- and illness-specific needs. The adaptive nature of the intervention ensures that it meets caregiver needs for that specific instance, flexibly changing for individuals over time in response to each intervention trigger. RE-PACT is also designed to acknowledge that respiratory illness in severe CP is driven by both respiratory and nonrespiratory comorbid and social conditions [11,46] (eg, neuromuscular weakness, seizures, dysphagia, feeding intolerance, health system navigation barriers, and coordination problems).

By conducting successively larger waves of the RE-PACT protocol, we expect to produce a final high-quality protocol that has been developed sufficiently to support the implementation of a large-scale multisite clinical efficacy trial.

### Limitations

This study has several limitations. Although we anticipate achieving a feasible, acceptable, and high-fidelity protocol by the end of the third wave, it is possible that some challenges may remain. We anticipate being underpowered to assess intervention efficacy. Despite the randomized design, allocation concealment is not possible. As randomization occurs at the level of the family, inadvertent intervention contamination to nonintervention patients in the same clinical program may occur. This risk will be minimized by having research staff (not clinical staff) manage action planning and SMS text messaging procedures. In future studies, we will consider alternative designs (such as a stepped wedge trial), randomizing at the clinic level to avoid this threat. Threats to external validity will reflect the relatively narrow population of families recruited from 2 complex care programs. Although it is a strength that the intervention will be conducted in English and Spanish, future expansion to populations of children with CP outside of complex care programs and from more geographically and culturally diverse settings will be helpful. As research continues, it will be important to examine whether this intervention design, which relies in part on the use of digital technology, addresses disparities in access to care and inequities in outcomes.

### Conclusions

Despite the limitations, our pilot RE-PACT intervention represents an innovative and promising strategy to reduce severe respiratory illness among children with severe CP. RE-PACT operationalizes universal action planning, mobile SMS text messaging, and a JIT adaptive rapid clinical response to deliver timely customized care to families of children with severe CP. This protocol describes detailed methods to assess intervention feasibility, acceptability, and fidelity. This line of research may be relevant to other researchers and health care providers who wish to adopt a similar early intervention strategy for patients with chronic and complex conditions at high risk of future hospitalization.

## Abbreviations

ACTIV: Assessing Confidence at Times of Increased Vulnerability
ANCOVA: analysis of covariance
COM-B: capability, opportunity, motivation, and behavior
CP: cerebral palsy
ED: emergency department
GMFCS: Gross Motor Function Classification System
*ICD-10*: *International Classification of Diseases, Tenth Revision*
JIT: just-in-time
mHealth: mobile health
NB: negative binomial
PACT: Plans for Action and Care Transitions
RE-PACT: Respiratory Exacerbation–Plans for Action and Care Transitions
REDCap: Research Electronic Data Capture
UCLA: University of California, Los Angeles
UW: University of Wisconsin–Madison

## Data Availability

The data sets generated and analyzed during this study are available from the corresponding author on reasonable request.

## References

[1] Boel L, Pernet K, Toussaint M, Ides K, Leemans G, Haan J, van Hoorenbeeck K, Verhulst S (2019). Respiratory morbidity in children with cerebral palsy: an overview. Dev Med Child Neurol.

[2] Fitzgerald DA, Follett J, van Asperen PP (2009). Assessing and managing lung disease and sleep disordered breathing in children with cerebral palsy. Paediatr Respir Rev.

[3] Murphy N, Such-Neibar T (2003). Cerebral palsy diagnosis and management: the state of the art. Curr Probl Pediatr Adolesc Health Care.

[4] Seddon PC, Khan Y (2003). Respiratory problems in children with neurological impairment. Arch Dis Child.

[5] Reid SM, Carlin JB, Reddihough DS (2012). Survival of individuals with cerebral palsy born in Victoria, Australia, between 1970 and 2004. Dev Med Child Neurol.

[6] Himmelmann K, Sundh V (2015). Survival with cerebral palsy over five decades in western Sweden. Dev Med Child Neurol.

[7] Centers for Disease ControlPrevention (CDC) (2004). Economic costs associated with mental retardation, cerebral palsy, hearing loss, and vision impairment--United States, 2003. MMWR Morb Mortal Wkly Rep.

[8] Murphy NA, Hoff C, Jorgensen T, Norlin C, Young PC (2006). Costs and complications of hospitalizations for children with cerebral palsy. Pediatr Rehabil.

[9] Meehan E, Reid SM, Williams K, Freed GL, Babl FE, Sewell JR, Rawicki B, Reddihough DS (2015). Tertiary paediatric emergency department use in children and young people with cerebral palsy. J Paediatr Child Health.

[10] Meehan E, Reid S, Williams K, Freed GL, Sewell JR, Vidmar S, Donath S, Reddihough DS (2017). Hospital admissions in children with cerebral palsy: a data linkage study. Dev Med Child Neurol.

[11] Blackmore AM, Bear N, Blair E, Langdon K, Moshovis L, Steer K, Wilson AC (2018). Predicting respiratory hospital admissions in young people with cerebral palsy. Arch Dis Child.

[12] Katz RT (2003). Life expectancy for children with cerebral palsy and mental retardation: implications for life care planning. NeuroRehabilitation.

[13] Raina P, O'Donnell M, Rosenbaum P, Brehaut J, Walter SD, Russell D, Swinton M, Zhu B, Wood E (2005). The health and well-being of caregivers of children with cerebral palsy. Pediatrics.

[14] Hayles E, Jones A, Harvey D, Plummer D, Ruston S (2015). Delivering healthcare services to children with cerebral palsy and their families: a narrative review. Health Soc Care Community.

[15] Jones M, Morgan E, Shelton JE (2007). Primary care of the child with cerebral palsy: a review of systems (part II). J Pediatr Health Care.

[16] Meehan E, Freed GL, Reid SM, Williams K, Sewell JR, Rawicki B, Reddihough DS (2015). Tertiary paediatric hospital admissions in children and young people with cerebral palsy. Child Care Health Dev.

[17] Coller RJ, Nelson BB, Klitzner TS, Saenz AA, Shekelle PG, Lerner CF, Chung PJ (2017). Strategies to reduce hospitalizations of children with medical complexity through complex care: expert perspectives. Acad Pediatr.

[18] Coller RJ, Klitzner TS, Saenz AA, Lerner CF, Nelson BB, Chung PJ (2015). The medical home and hospital readmissions. Pediatrics.

[19] Berry J, Ziniel S, Freeman L, Kaplan W, Antonelli R, Gay J, Coleman EA, Porter S, Goldmann D (2013). Hospital readmission and parent perceptions of their child's hospital discharge. Int J Qual Health Care.

[20] Coller RJ, Nelson BB, Sklansky DJ, Saenz AA, Klitzner TS, Lerner CF, Chung PJ (2014). Preventing hospitalizations in children with medical complexity: a systematic review. Pediatrics.

[21] Nelson BB, Coller RJ, Saenz AA, Chung PJ, Kaplan A, Lerner CF, Klitzner TS (2016). How avoidable are hospitalizations for children with medical complexity? understanding parent perspectives. Acad Pediatr.

[22] Zemek RL, Bhogal SK, Ducharme FM (2008). Systematic review of randomized controlled trials examining written action plans in children: what is the plan?. Arch Pediatr Adolesc Med.

[23] Gibson PG, Powell H (2004). Written action plans for asthma: an evidence-based review of the key components. Thorax.

[24] Bhogal S, Zemek R, Ducharme FM (2006). Written action plans for asthma in children. Cochrane Database Syst Rev.

[25] Coleman EA, Roman SP, Hall KA, Min SJ (2015). Enhancing the care transitions intervention protocol to better address the needs of family caregivers. J Healthc Qual.

[26] Coleman EA, Smith JD, Frank JC, Min SJ, Parry C, Kramer AM (2004). Preparing patients and caregivers to participate in care delivered across settings: the care transitions intervention. J Am Geriatr Soc.

[27] Coleman EA, Parry C, Chalmers S, Min SJ (2006). The care transitions intervention: results of a randomized controlled trial. Arch Intern Med.

[28] Coller R, Klitzner T, Lerner C, Nelson BB, Thompson LR, Zhao Q, Saenz AA, Ia S, Flores-Vazquez J, Chung PJ (2018). Complex care hospital use and postdischarge coaching: a randomized controlled trial. Pediatrics.

[29] Coller RJ, Lerner CF, Berry JG, Klitzner TS, Allshouse C, Warner G, Nacht CL, Thompson LR, Eickhoff J, Ehlenbach ML, Bonilla AJ, Venegas M, Garrity BM, Casto E, Bowe T, Chung PJ (2021). Linking parent confidence and hospitalization through mobile health: a multisite pilot study. J Pediatr.

[30] Coller RJ, Lerner CF, Chung PJ, Klitzner TS, Cushing CC, Warner G, Nacht CL, Thompson LR, Eickhoff J, Ehlenbach ML, Garrity BM, Bowe T, Berry JG (2022). Caregiving and confidence to avoid hospitalization for children with medical complexity. J Pediatr.

[31] Barreda CB, Ehlenbach ML, Nackers A, Kelly MM, Shadman KA, Sklansky DJ, Edmonson MB, Zhao Q, Warner G, Coller RJ (2021). Complex care program enrollment and change in ED and hospital visits from medical device complications. Pediatr Qual Saf.

[32] McDowell B (2008). The gross motor function classification system--expanded and revised. Dev Med Child Neurol.

[33] Nahum-Shani I, Smith SN, Spring BJ, Collins LM, Witkiewitz K, Tewari A, Murphy SA (2018). Just-in-time adaptive interventions (JITAIs) in mobile health: key components and design principles for ongoing health behavior support. Ann Behav Med.

[34] Nakamura MM, Zaslavsky AM, Toomey SL, Petty CR, Bryant MC, Geanacopoulos AT, Jha AK, Schuster MA (2017). Pediatric readmissions after hospitalizations for lower respiratory infections. Pediatrics.

[35] Leyenaar JK, Lagu T, Shieh MS, Pekow PS, Lindenauer PK (2014). Management and outcomes of pneumonia among children with complex chronic conditions. Pediatr Infect Dis J.

[36] Bradley JS, Byington CL, Shah SS, Alverson B, Carter ER, Harrison C, Kaplan SL, Mace SE, McCracken GH, Moore MR, St Peter SD, Stockwell JA, Swanson JT (2011). The management of community-acquired pneumonia in infants and children older than 3 months of age: clinical practice guidelines by the Pediatric Infectious Diseases Society and the Infectious Diseases Society of America. Clin Infect Dis.

[37] Keyworth C, Epton T, Goldthorpe J, Calam R, Armitage CJ (2020). Acceptability, reliability, and validity of a brief measure of capabilities, opportunities, and motivations ("COM-B"). Br J Health Psychol.

[38] Michie S, van Stralen MM, West R (2011). The behaviour change wheel: a new method for characterising and designing behaviour change interventions. Implement Sci.

[39] Coleman EA, Ground KL, Maul A (2015). The Family Caregiver Activation in Transitions (FCAT) tool: a new measure of family caregiver self-efficacy. Jt Comm J Qual Patient Saf.

[40] Luszczynska A, Scholz U, Schwarzer R (2005). The general self-efficacy scale: multicultural validation studies. J Psychol.

[41] Measures: family experience with care coordination measure set (FECC). Agency for Healthcare Research and Quality.

[42] Rosenbaum PL, Palisano RJ, Bartlett DJ, Galuppi BE, Russell DJ (2008). Development of the gross motor function classification system for cerebral palsy. Dev Med Child Neurol.

[43] Palisano RJ, Rosenbaum P, Bartlett D, Livingston MH (2008). Content validity of the expanded and revised gross motor function classification system. Dev Med Child Neurol.

[44] Brannan AM, Athay MM, de Andrade AR (2012). Measurement quality of the Caregiver Strain Questionnaire-Short Form 7 (CGSQ-SF7). Adm Policy Ment Health.

[45] Arroll B, Goodyear-Smith F, Crengle S, Gunn J, Kerse N, Fishman T, Falloon K, Hatcher S (2010). Validation of PHQ-2 and PHQ-9 to screen for major depression in the primary care population. Ann Fam Med.

[46] Gibson N, Blackmore AM, Chang AB, Cooper MS, Jaffe A, Kong WR, Langdon K, Moshovis L, Pavleski K, Wilson AC (2021). Prevention and management of respiratory disease in young people with cerebral palsy: consensus statement. Dev Med Child Neurol.

